# Quality of Vitamin K Antagonist Control and 1-Year Outcomes in Patients with Atrial Fibrillation: A Global Perspective from the GARFIELD-AF Registry

**DOI:** 10.1371/journal.pone.0164076

**Published:** 2016-10-28

**Authors:** Sylvia Haas, Hugo ten Cate, Gabriele Accetta, Pantep Angchaisuksiri, Jean-Pierre Bassand, A. John Camm, Ramon Corbalan, Harald Darius, David A. Fitzmaurice, Samuel Z. Goldhaber, Shinya Goto, Barry Jacobson, Gloria Kayani, Lorenzo G. Mantovani, Frank Misselwitz, Karen Pieper, Sebastian M. Schellong, Janina Stepinska, Alexander G. G. Turpie, Martin van Eickels, Ajay K. Kakkar, for the GARFIELD-AF Investigators

**Affiliations:** 1 Formerly Klinikum rechts der Isar, Technical University of Munich, Munich, Germany; 2 Department of Internal Medicine, Cardiovascular Research Institute Maastricht, Maastricht, The Netherlands; 3 Thrombosis Research Institute, London, United Kingdom; 4 Department of Medicine, Ramathibodi Hospital, Mahidol University, Bangkok, Thailand; 5 Department of Cardiology, EA 3920, University of Besançon, Besançon, France; 6 Division of Cardiovascular Sciences, St George’s University of London, London, United Kingdom; 7 Department of Cardiology, Catholic University School of Medicine, Santiago, Chile; 8 Department of Cardiology, Vascular Medicine and Intensive Care Medicine, Vivantes Neukoelln Medical Centre, Berlin, Germany; 9 Department of Primary Care Clinical Sciences, University of Birmingham, Edgbaston, Birmingham, United Kingdom; 10 Division of Cardiovascular Medicine, Brigham and Women’s Hospital and Harvard Medical School, Boston, MA, United States of America; 11 Department of Medicine (Cardiology), Tokai University School of Medicine, Isehara, Japan; 12 Department of Hematology and Molecular Medicine, Johannesburg Hospital, University of the Witwatersrand, Johannesburg, South Africa; 13 Center for Public Health Research (CESP), University of Milano-Bicocca, Milan, Italy; 14 Bayer Pharma AG, Berlin, Germany; 15 Duke Clinical Research Institute, Durham, NC, United States of America; 16 Internal Medicine, Dresden-Friedrichstadt Hospital, Dresden, Germany; 17 Intensive Cardiac Therapy Clinic, Institute of Cardiology, Warsaw, Poland; 18 Department of Medicine, McMaster University, Hamilton, Canada; 19 University College London, London, United Kingdom; Institut d'Investigacions Biomediques de Barcelona, SPAIN

## Abstract

**Aims:**

Vitamin K antagonists (VKAs) need to be individually dosed. International guidelines recommend a target range of international normalised ratio (INR) of 2.0–3.0 for stroke prevention in atrial fibrillation (AF). We analysed the time in this therapeutic range (TTR) of VKA-treated patients with newly diagnosed AF in the ongoing, global, observational registry GARFIELD-AF. Taking TTR as a measure of the quality of patient management, we analysed its relationship with 1-year outcomes, including stroke/systemic embolism (SE), major bleeding, and all-cause mortality.

**Methods and Results:**

TTR was calculated for 9934 patients using 136,082 INR measurements during 1-year follow-up. The mean TTR was 55.0%; values were similar for different VKAs. 5851 (58.9%) patients had TTR<65%; 4083 (41.1%) TTR≥65%. The proportion of patients with TTR≥65% varied from 16.7% in Asia to 49.4% in Europe. There was a 2.6-fold increase in the risk of stroke/SE, 1.5-fold increase in the risk of major bleeding, and 2.4-fold increase in the risk of all-cause mortality with TTR<65% versus ≥65% after adjusting for potential confounders. The population attributable fraction, i.e. the proportion of events attributable to suboptimal anticoagulation among VKA users, was 47.7% for stroke/SE, 16.7% for major bleeding, and 45.4% for all-cause mortality. In patients with TTR<65%, the risk of first stroke/SE was highest in the first 4 months and decreased thereafter (test for trend, p = 0.021). In these patients, the risk of first major bleed declined during follow-up (p = 0.005), whereas in patients with TTR≥65%, the risk increased over time (p = 0.027).

**Conclusion:**

A large proportion of patients with AF had poor VKA control and these patients had higher risks of stroke/SE, major bleeding, and all-cause mortality. Our data suggest that there is room for improvement of VKA control in routine clinical practice and that this could substantially reduce adverse outcomes.

**Trial Registration:**

ClinicalTrials.gov NCT01090362

## Introduction

Vitamin K antagonists (VKAs) had been the only recommended anticoagulants for stroke prevention in patients with atrial fibrillation (AF) before non-vitamin K antagonist oral anticoagulants (NOACs) were developed for this indication [1]. Despite the beneficial benefit/risk ratio of NOACs, VKAs have remained a frequently used therapy in clinical routine. The Global Anticoagulant Registry in the FIELD–Atrial Fibrillation (GARFIELD-AF) is an observational study of patients newly diagnosed with non-valvular AF evaluating the management of these patients in clinical routine worldwide [2]. The registry started in 2009, at the end of the VKA-only era, providing insights into changes in treatment patterns over time.

An analysis of the first 10,614 patients included in the GARFIELD-AF registry has shown that the patients’ stroke risk profile does not always match the prescribing pattern of anticoagulants [3]. The 2013 publication describes the baseline characteristics and initial therapeutic management of patients with non-valvular AF, with and without antithrombotic treatment, and indicates that anticoagulant drugs are frequently not being used according to stroke risk scores and guidelines, with overuse in patients at low risk and underuse in those at high risk of stroke. This is a crucial finding for the use of VKAs since these anticoagulants have a narrow therapeutic window and need to be individually dosed based on international normalised ratio (INR) control. According to international guidelines, the optimal INR range to minimise ischaemic stroke and bleeding is between 2.0–3.0 [4, 5]. Various methods are used to describe the quality of INR control, e.g., proportion of time in therapeutic range (TTR), frequency in therapeutic range (FIR), and levels of anticoagulation in narrow INR bands of 0.5. Recently, we showed that TTR and FIR are not equivalent and should not be used interchangeably [6]. Our earlier analyses also suggested that TTR may be preferable to FIR because it takes into account the time between INR readings. However, both methods refer to a predefined target range of INR and so information about over- or underanticoagulation is not obtained.

In this paper, we analyse the TTR of VKA-treated patients enrolled prospectively in the GARFIELD-AF registry and analyse the relationship between TTR and 1-year outcomes (stroke/systemic embolism [SE], major bleeding, and all-cause mortality).

## Methods

### Study design

GARFIELD-AF is a worldwide observational prospective registry of adults with newly diagnosed non-valvular AF [2]. Women and men aged ≥18 years with a diagnosis of non-valvular AF within the past 6 weeks and at least one additional investigator-defined risk factor for stroke are eligible for enrolment, regardless of therapy. Patients with a transient reversible cause of AF and those for whom follow-up is unlikely are excluded. Patients are enrolled from 2010 to 2016.

This paper reports 1-year follow-up data for prospective patients enrolled from May 2010 to September 2015 treated with VKA (with or without antiplatelet therapy). The data were extracted from the study database on 28 July 2016.

### Ethics statement

All patients provided written informed consent to participate. Independent ethics committee and hospital-based institutional review board approvals were obtained, as necessary, for the registry protocol. A list of central ethics committees and regulatory authorities that provided approval can be found in S2 File. Additional approvals were obtained from individual study sites. The registry is being conducted in accordance with the principles of the Declaration of Helsinki, local regulatory requirements, and the International Conference on Harmonisation-Good Pharmacoepidemiological and Clinical Practice Guidelines.

### Data collection

Data were collected using an electronic case report form (eCRF) and captured by trained personnel. The eCRF was designed by Dendrite Clinical Systems Ltd, Henley-on-Thames, UK, which is also responsible for ongoing database program management. Outcome events were reported by investigators, after review of patient notes and clinical records, and audited using a combination of remote electronic monitoring and more conventional onsite monitoring (including source data verification on 20% of cases) [2].

### Statistical analysis

#### INR readings and TTR

INR readings during the first year of follow-up were included in the analysis. TTR was estimated between two consecutive INR readings only if the interval did not exceed 90 days. Implausible INR values of less than 0.8 or greater than 20 were excluded. Patients on VKA treatment at enrolment, but with fewer than three readings during the follow-up, were excluded from the analysis. Patient-level TTR was estimated by linear interpolation according to Rosendaal et al. [7], using 2.0–3.0 as the target INR range. TTR was estimated using INR readings until discontinuation or interruption of VKA, an outcome event, or the end of follow-up. Thus, INR values after a stroke/SE were not used to study the relationship between TTR and stroke/SE, and INR values after a major bleed were not used to study the relationship between TTR and major bleeding.

#### Baseline patient characteristics

Baseline patient characteristics were analysed for the total population and by level of TTR, using the cut-off of 65%. TTR<65% has been defined by the UK National Institute for Health and Care Excellence (NICE) to indicate poor VKA control [8]. Counts and percentages are reported for categorical variables; means and standard deviations (SDs) are reported for continuous variables.

#### TTR by type of VKA and by concomitant antiplatelet therapy

The types of VKA (warfarin, acenocoumarol, phenprocoumon and other) received by patients at enrolment are collected. For the category of ‘other’ VKA therapies, the drug names are not collected. The types of VKA are reported as counts and percentages. Mean, SD, median, and interquartile range (IQR) were estimated to describe the distribution of TTR by type of VKA and by concomitant antiplatelet therapy.

#### Distribution of INR values

The distribution of INR values is described by counts and percentages below, within, and above the therapeutic range, and by the mean, SD, median, and IQR.

#### Clinical outcomes

Stroke/SE, major bleeding, and all-cause mortality events occurring during the first year of follow-up are described using the number of events, the population at risk at the beginning of the follow-up period, and rate. Only the first occurrence of each event was taken into account. Haemorrhagic strokes were counted as a major bleed event and as a stroke event. Person-time event rates (per 100 person-years) by the TTR cut-off of 65% were estimated using a Poisson model with the number of events as the dependent variable and the log of person-time as an offset.

Hazard ratios (HRs) were estimated for TTR<65% vs TTR≥65% (reference group) using a Cox model. The proportional hazards assumption was assessed visually using a plot of the survivor function over time by TTR level. HRs were controlled for the following potential confounders: age group (≤64, 65–69, 70–74, ≥75 years), gender, smoking (no, ex, current), congestive heart failure, vascular disease, moderate-to-severe chronic kidney disease, diabetes mellitus, hypertension, previous stroke (not included in the model for major bleeding events), previous bleeding (not included in the model for stroke/SE), antiplatelet treatment, type of AF, and area (Europe, Asia, other countries).

A patient may be classified in different groups (TTR≥65% or TTR<65%) for different clinical outcomes, since INR readings after the event were not included when estimating TTR. Strokes occurring after a major bleed were included in the analysis of strokes; major bleeds after a stroke event were included in the analysis of major bleeding; and deaths after a stroke or major bleed were included in the mortality analysis. The estimation of TTR was censored when there was an interruption of VKA treatment; however, the time to event in the Cox model was not censored for an interruption of VKA treatment.

The incidence rates described above were analysed by 4-month intervals as well. The overall TTR values used in the main analyses were also used to define the TTR group for each 4-month interval. The 4-month rates of events were compared with the overall rates using the ratio between the observed number of events in the period and the expected number of events obtained by applying the overall rate to the period. The Poisson trend statistic was used to assess the trends over time.

#### Sensitivity analysis

Estimation of TTR and analysis of outcomes were repeated, excluding INR readings and events during the first 3 months of treatment for patients with three or more readings during the last 9 months.

#### Missing values

In descriptive statistics, for a single variable we excluded all patients for which the variable was missing (available-case analysis). HRs were estimated using a proportional hazards Cox model after multiple imputation by the Multiple Imputation by Chained Equations (MICE) algorithm.

#### Statistical software

Analyses were double performed using SAS, release 9.4 (SAS Institute, Cary, NC, USA) and Stata, version 14 (StataCorp, College Station, TX, USA).

## Results

### Baseline patient characteristics

In total, 39,898 patients were enrolled in GARFIELD-AF. Of the 39,368 patients with data on antithrombotic treatment at baseline, 16,852 (42.8%) were treated with VKAs (with or without antiplatelet therapy). Among the VKA-treated patients, 6440 (38.2%) had <3 INR readings and were excluded from the analysis. Of the 10,412 remaining patients, 478 (4.6%) who had INR readings with a gap of >90 days were excluded, leaving 9934 patients (58.9% of VKA-treated patients) who had at least three INR readings with an interval not exceeding 90 days, with a total of 136,082 INR measurements. Among the patients included in the analysis, 82.1% had 6+ INR readings. The number of patients enrolled in each of the 35 countries participating in GARFIELD-AF is shown in S1 Table.

Using INR 2.0–3.0 as the target range, 5851 patients (58.9%) had a TTR of <65% and 4083 patients (41.1%) had a TTR of ≥65%. Baseline characteristics of the overall population and according to TTR level (<65% vs ≥65%) are shown in Table 1. A greater proportion of the TTR<65% group had a body mass index (BMI) of <25 kg/m^2^ compared with the TTR≥65% group (32.6% vs 25.9%). Conversely, a greater proportion of the TTR≥65% group had a BMI of 30–<40 kg/m^2^ (31.0% vs 26.4% for the <65% group). The proportion of patients with TTR≥65% varied from 16.7% in Asia to 49.4% in Europe (Table 2).

**Table 1:** Baseline characteristics of patients according to proportion of time in therapeutic range.

	TTR<65% (N = 5851; 58.9%)		TTR≥65% (N = 4083; 41.1%)		Total (N = 9934; 100%)	
Age, mean (SD), years	70.7 (10.6)		71.9 (9.7)		71.2 (10.2)	
Age group, n/N, %						
65–74 years	2029/5851	34.7	1430/4083	35.0	3459/9934	34.8
≥75 years	2343/5851	40.0	1786/4083	43.7	4129/9934	41.6
Women, n/N, %	2649/5851	45.3	1791/4083	43.9	4440/9934	44.7
BMI category, n/N, %						
<19 kg/m^2^	109/4594	2.4	40/3137	1.3	149/7731	1.9
19–<25 kg/m^2^	1390/4594	30.3	771/3137	24.6	2161/7731	28.0
25–<30 kg/m^2^	1715/4594	37.3	1245/3137	39.7	2960/7731	38.3
30–<40 kg/m^2^	1214/4594	26.4	973/3137	31.0	2187/7731	28.3
≥40 kg/m^2^	166/4594	3.6	108/3137	3.4	274/7731	3.5
Medical history, n/N, %						
Congestive heart failure	1189/5851	20.3	673/4083	16.5	1862/9934	18.7
History of hypertension	4574/5842	78.3	3283/4073	80.6	7857/9915	79.2
Diabetes mellitus	1490/5851	25.5	888/4083	21.7	2378/9934	23.9
Prior stroke/transient ischaemic attack	751/5851	12.8	583/4083	14.3	1334/9934	13.4
Vascular disease*	814/5842	13.9	608/4077	14.9	1422/9919	14.3
Chronic kidney disease (Grade ≥3)^†^	749/5851	12.8	522/4083	12.8	1271/9934	12.8
History of bleeding	121/5838	2.1	70/4077	1.7	191/9915	1.9
Alcohol consumption, n/N, %						
Abstinent	2585/4943	52.3	1544/3346	46.1	4129/8289	49.8
Light	1701/4943	34.4	1427/3346	42.6	3128/8289	37.7
Moderate	521/4943	10.5	323/3346	9.7	844/8289	10.2
Heavy	136/4943	2.8	52/3346	1.6	188/8289	2.3
Smoker, n/N, %						
No	3329/5277	63.1	2332/3671	63.5	5661/8948	63.3
Ex-smoker	1411/5277	26.7	1055/3671	28.7	2466/8948	27.6
Current smoker	537/5277	10.2	284/3671	7.7	821/8948	9.2

*Peripheral artery disease or coronary artery disease with a history of acute coronary syndromes.

^†^Renal function was assessed according to the National Kidney Foundation’s Kidney Disease Outcomes Quality Initiative classification by investigators at baseline.

BMI, body mass index; SD, standard deviation; TTR, time in therapeutic range.

**Table 2:** Proportion of patients with TTR <65% and ≥65% in different geographic regions.

	TTR<65% (n/N, %)		TTR≥65% (n/N, %)	
Europe	3462/6840	50.6	3378/6840	49.4
North America	119/220	54.1	101/220	45.9
Latin America	461/588	78.4	127/588	21.6
Asia	1581/1899	83.3	318/1899	16.7
Rest of the world	228/387	58.9	159/387	41.1

TTR, time in therapeutic range.

Europe: Austria, Belgium, Czech Republic, Denmark, Finland, France, Germany, Hungary, Italy, Netherlands, Norway, Poland, Russia, Spain, Sweden, Switzerland, Ukraine, UK; North America: Canada, USA; Latin America: Argentina, Brazil, Chile, Mexico; Asia: China, India, Japan, Korea, Singapore, Thailand, Turkey, UAE; rest of the world: Australia, Egypt, South Africa.

### VKA control

Overall, the mean (SD) INR was 2.4 (0.9) and the median (IQR) was 2.3 (1.8 to 2.8). Of the total INR values, 51.4% were in the therapeutic range of 2.0–3.0, 32.1% were below, and 16.5% were above this range.

Warfarin was the most frequently used VKA at enrolment (66.7% of patients), followed by acenocoumarol (24.0% of patients; Table 3). Of the patients receiving ‘other’ VKA therapy, the majority were from France (191/231, 82.7%), where published reports indicate that fluindione is the most frequently used VKA [9]. The mean TTR for the total study group was 55.0%. The mean TTR for patients receiving warfarin was 55.4% (median: 60.8%). Patients on acenocoumarol at enrolment had the lowest TTR (mean: 52.9%; median: 54.7%). The mean TTR was similar for patients on VKA only and for those on VKA with antiplatelet therapy (Table 4). The mean TTR values were <65% for all types of VKA regardless of whether patients received concomitant antiplatelet therapy.

**Table 3:** Proportion of time in therapeutic range (%) for patients on different vitamin K antagonists*.

VKA therapy	n (%)	Mean	SD	Median	Interquartile range
Warfarin	6513 (66.7)	55.4	28.4	60.8	35.7 to 77.6
Acenocoumarol	2345 (24.0)	52.9	23.7	54.7	37.4 to 70.4
Phenprocoumon	672 (6.9)	56.2	26.7	57.6	37.3 to 77.3
Other	231 (2.4)	59.2	28.1	65.6	38.9 to 80.4

*N = 9934; data missing for 173 patients.

SD, standard deviation; VKA, vitamin K antagonist.

**Table 4:** Proportion of time in therapeutic range (%) for patients on vitamin K antagonists with and without concomitant antiplatelet therapy.

Therapy	n (%)	Mean	SD	Median	Interquartile range
VKA	7752 (78.0)	55.5	27.1	59.4	37.6 to 76.0
VKA+AP	2182 (22.0)	53.3	27.9	56.7	33.4 to 75.3

AP, antiplatelet; SD, standard deviation; VKA, vitamin K antagonist.

### Clinical outcomes

**Fig 1:**
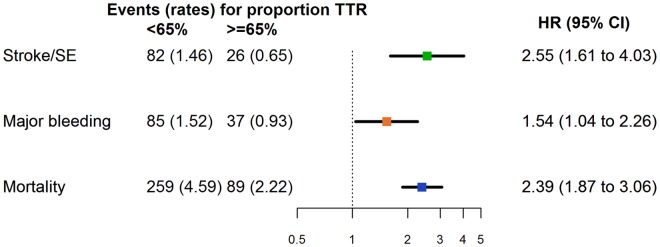
Incidence rates and adjusted hazard ratios for 1-year clinical outcomes according to proportion of time in therapeutic range. Reference group: TTR≥65%. Incidence rates are per 100 person-years. CI, confidence interval; HR, hazard ratio; SE, systemic embolism; TTR, time in therapeutic range. HRs were controlled for the following potential confounders: age group (≤64, 65–69, 70–74, ≥75 years), gender, smoking (no, ex, current), congestive heart failure, vascular disease, moderate-to-severe chronic kidney disease, diabetes mellitus, hypertension, previous stroke (not included in the model for major bleeding events), previous bleeding (not included in the model for stroke/SE), antiplatelet treatment, type of atrial fibrillation, and area (Europe, Asia, other countries).

The rate of stroke/SE during 1-year follow-up was 1.46 (95% confidence interval [CI] 1.18 to 1.82) per 100 person-years for patients with TTR<65% and 0.65 (0.44 to 0.96) per 100 person-years for patients with TTR≥65% (Fig 1). The rate of major bleeding was 1.52 (1.23 to 1.88) per 100 person-years for patients with TTR<65% and 0.93 (0.67 to 1.28) per 100 person-years for patients with TTR≥65% (Fig 1). The rate of all-cause mortality was 4.59 (4.06 to 5.18) per 100 person-years for patients with TTR<65% and 2.22 (1.80 to 2.73) per 100 person-years for patients with TTR≥65% (Fig 1). For the first major bleeding events, 14 (11.5%) were followed by a first stroke.

HRs showed a 2.6-fold increase in the risk of stroke/SE with a TTR of <65% compared with ≥65% (Fig 1 and Table 5). TTR of <65% was also associated with a 1.5-fold increase in the risk of major bleeding and a 2.4-fold increase in the risk of all-cause mortality. The population attributable fraction, i.e. the proportion of events attributable to suboptimal anticoagulation among VKA users, was 47.7% for stroke/SE, 16.7% for major bleeding, and 45.4% for all-cause mortality. The sensitivity analysis, excluding INR readings and events during the first 3 months of treatment, showed that the increased risks of stroke/SE, major bleeding and all-cause mortality associated with TTR<65% prevailed (Table 5).

**Table 5:** Adjusted hazard ratios for 1-year clinical outcomes by proportion of time in therapeutic range for the main analysis and sensitivity analysis (excluding international normalised ratio readings and events during the first 3 months of treatment).

		Main analysis				Sensitivity analysis			
		Population at risk (N)	Events	HR	95% CI	Population at risk (N)	Events	HR	95% CI
Stroke/SE	TTR≥65%	4080	26	1	-	3967	24	1	-
	TTR<65%	5844	82	2.55	1.61 to 4.03	4211	48	2.35	1.38 to 4.00
Major bleeding	TTR≥65%	4081	37	1	-	3974	43	1	-
	TTR<65%	5842	85	1.54	1.04 to 2.26	4206	45	1.31	0.83 to 2.06
All-cause mortality	TTR≥65%	4083	89	1	-	3968	63	1	-
	TTR<65%	5851	259	2.39	1.87 to 3.06	4218	132	2.27	1.74 to 2.97

CI, confidence interval; HR, hazard ratio; SE, systemic embolism; TTR, time in therapeutic range.

HRs were controlled for the following potential confounders: age group (≤64, 65–69, 70–74, ≥75 years), gender, smoking (no, ex, current), congestive heart failure, vascular disease, moderate-to-severe chronic kidney disease, diabetes mellitus, hypertension, previous stroke (not included in the model for major bleeding events), previous bleeding (not included in the model for stroke/SE), antiplatelet treatment, type of atrial fibrillation, and area (Europe, Asia, other countries).

**Fig 2:**
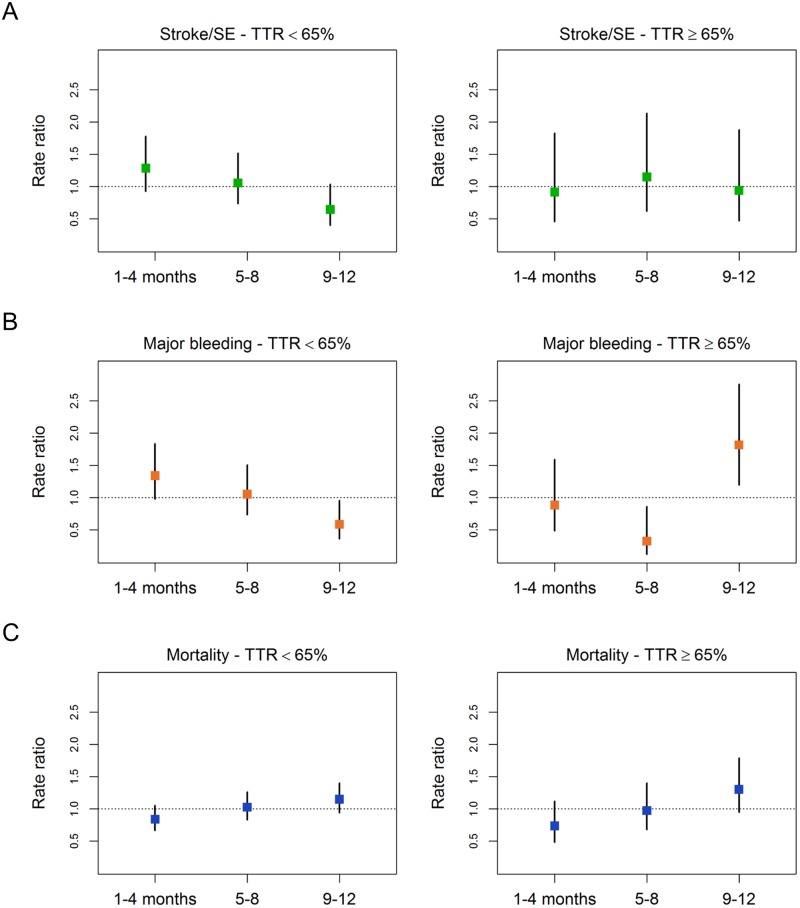
Four-month rate ratios by proportion of time in therapeutic range for (A) stroke/systemic embolism, (B) major bleeding, and (C) all-cause mortality. SE, systemic embolism; TTR, time in therapeutic range.

The risk of the first stroke/SE during this follow-up period was highest in the first 4 months of follow-up and decreased over time in patients with TTR<65%, whereas no change in risk of stroke/SE was seen in patients with TTR≥65% over time (Table 6, Fig 2A and S2 Table; test for trend, TTR<65% p = 0.021, TTR≥65% p = 0.999, overall p = 0.045). The types of stroke are listed in S3 Table, which shows that the risk of stroke in the first 4 months was clearly dominated by ischaemic events in patients with TTR<65%, whereas the number of haemorrhagic strokes did not change over time regardless of whether TTR was <65% or ≥65%. The risk of first major bleed declined over time in patients with TTR<65%, while it increased in patients with TTR≥65% (Table 6, Fig 2B and S2 Table; test for trend, TTR<65% p = 0.005, TTR≥65% p = 0.027). Overall, the risk of first major bleed in all patients did not show a trend during the follow-up period (Table 6 and S2 Table; test for trend, p = 0.267). The risk of all-cause mortality during the follow-up period increased in patients with TTR<65% and in those with TTR≥65% (Table 6, Fig 2C and S2 Table; test for trend, TTR<65% p = 0.047, TTR≥65% p = 0.027, overall p = 0.005).

**Table 6:** Four-month event rates in patients with proportion of time in therapeutic range <65% and ≥65%.

		TTR<65%		TTR≥65%		Total	
		Events	Rate, per 100 person-years (95% CI)	Events	Rate, per 100 person-years (95% CI)	Events	Rate, per 100 person-years (95% CI)
**Stroke/SE**	1^st^ to 4^th^ months	36	1.88 (1.35 to 2.60)	8	0.59 (0.30 to 1.19)	44	1.35 (1.00 to 1.81)
	5^th^ to 8^th^ months	29	1.54 (1.07 to 2.22)	10	0.75 (0.40 to 1.39)	39	1.21 (0.89 to 1.66)
	9^th^ to 12^th^ months	17	0.94 (0.59 to 1.52)	8	0.61 (0.31 to 1.22)	25	0.80 (0.54 to 1.19)
	Total	82	1.46 (1.18 to 1.82)	26	0.65 (0.44 to 0.96)	108	1.13 (0.93 to 1.36)
**Major bleeding**	1^st^ to 4^th^ months	39	2.03 (1.49 to 2.78)	11	0.82 (0.45 to 1.48)	50	1.53 (1.16 to 2.02)
	5^th^ to 8^th^ months	30	1.60 (1.12 to 2.28)	4	0.30 (0.11 to 0.80)	34	1.06 (0.76 to 1.48)
	9^th^ to 12^th^ months	16	0.89 (0.54 to 1.45)	22	1.68 (1.11 to 2.56)	38	1.22 (0.89 to 1.68)
	Total	85	1.52 (1.23 to 1.88)	37	0.93 (0.67 to 1.28)	122	1.27 (1.07 to 1.52)
**All-cause mortality**	1^st^ to 4^th^ months	74	3.84 (3.06 to 4.83)	22	1.63 (1.07 to 2.47)	96	2.93 (2.40 to 3.58)
	5^th^ to 8^th^ months	89	4.70 (3.82 to 5.78)	29	2.16 (1.50 to 3.11)	118	3.64 (3.04 to 4.36)
	9^th^ to 12^th^ months	96	5.27 (4.31 to 6.43)	38	2.89 (2.11 to 3.97)	134	4.27 (3.61 to 5.06)
	Total	259	4.59 (4.06 to 5.18)	89	2.22 (1.80 to 2.73)	348	3.61 (3.25 to 4.01)

CI, confidence interval; SE, systemic embolism; TTR, time in therapeutic range.

## Discussion

Contemporary, observational, worldwide data from 10,614 patients in the GARFIELD-AF registry, enrolled from 2009 to 2011 in 19 countries, have shown that anticoagulants are often overused in patients at low risk and underused in those at high risk of stroke [3]. VKAs remain a commonly prescribed anticoagulant therapy in patients with AF, even after the introduction of NOACs. This paper is the first publication of GARFIELD-AF analysing outcomes related to quality of VKA control. The analysis includes 9934 VKA-treated patients with at least three INR readings, out of a total of 39,898 patients recruited in GARFIELD-AF from 2010 to 2015 in 35 countries. All patients had newly diagnosed non-valvular AF and were followed up for 1 year.

European and American guidelines for stroke prevention in AF recommend an INR range of 2.0–3.0 for patients treated with VKA [4, 5], although country- and region-specific guidance may differ. The Japanese Circulation Society recommends an INR of 2.0–3.0 for all patients with AF except those aged ≥70 years, for whom 1.6–2.6 is recommended [10], which is supported by the Asia Pacific Heart Rhythm Society statement on antithrombotic therapy of patients with non-valvular AF [11]. The Federation of Dutch Thrombosis Services, until recently, defined the target INR range of 2.5–3.5 and the therapeutic range of 2.0–3.5 [12]. For the purposes of these analyses, we applied the target INR range of 2.0–3.0 and a cut-off TTR of 65% as the measure of the quality of patient management. In international guidelines, the cut-off of TTR varies between 60% and 70% [4, 10, 11], and NICE uses TTR of less than 65% as an indicator for poor control [8].

Real-world data from our study indicate that a TTR target of at least 65% is often not achieved in routine clinical practice, regardless of the type of VKA. For all VKAs used in GARFIELD-AF including warfarin, acenocoumarol, phenprocoumon, and others, the mean TTR values were below 65%. In addition, we observed marked regional variations in TTR, with the proportion of patients with TTR≥65% varying from 16.7% in Asia to 49.4% in Europe. The low proportion of patients with TTR≥65% in Asia is consistent with a previous analysis from GARFIELD-AF showing that patients in Asia had lower INR values than those in other regions of the world [13]. It may be that physicians in Asia target a lower INR than those in other regions (as per the Asia Pacific Heart Rhythm Society guidelines for patients aged ≥70 years [11]). Our analysis also showed that patients in Asia had a lower frequency of INR measurements.

Since TTR does not provide information about over- or underanticoagulation, additional information in this regard can be obtained by the percentages of INR values within, below, or above the target range of 2.0–3.0. Only 51.4% of all INR values measured were within range, whereas 32.1% were below and 16.5% above this range. Thus, underanticoagulation is more frequent than overanticoagulation in this global registry. This confirms the conclusion from a previous meta-analysis and meta-regression on quality of VKA control and outcomes in patients with AF where patients on VKA were frequently outside the target INR range and tended to be underanticoagulated rather than overanticoagulated [14].

As shown in S3 Table, there was a clear predominance of primary ischaemic strokes over primary intracerebral haemorrhages in both TTR groups, although this was much more pronounced in patients with TTR<65%. Suboptimal anticoagulation control determined by TTR<65% was associated with a predominance of ischaemic stroke events, especially in the first 4 months of follow-up. When all types of stroke were taken together, an increased risk of 1-year stroke/SE was observed with TTR<65%, compared with TTR≥65%. This trend was also apparent for major bleeding, and all-cause mortality, as shown in Fig 1. In our study, the population attributable fraction, i.e. the proportion of events attributable to suboptimal anticoagulation among VKA users, was 47.7% for stroke/SE, 16.7% for major bleeding, and 45.4% for all-cause mortality. An advantage of our analyses is that they reflect the use of VKAs in real life, where VKA treatment in patients with AF usually is not initiated with overlapping treatment with heparin to overcome the prothrombotic activity of VKA in the initial treatment period.

Putting these results into perspective, it is apparent from the literature that optimisation of VKA control has not improved over time. Hylek et al. [15] analysed the lowest effective intensity of prophylactic anticoagulation for patients with nonrheumatic AF and stated that tight control of anticoagulant therapy to maintain the INR between 2.0 and 3.0 is a better strategy than targeting lower, less effective levels of anticoagulation. More recently, Gallagher et al. [16] in a retrospective study of the medical records from 37,907 patients with AF from the UK General Practice Research Database found that TTR was a strong predictor of stroke. Another retrospective study based on Swedish registries, especially AuriculA, a quality register for AF and oral anticoagulation, was recently published [17]. In total, 40,449 patients with non-valvular AF started on warfarin were monitored until treatment cessation, death, or the end of the study. The authors concluded that well-managed warfarin therapy is associated with a low risk of complications and is still a valid alternative for prophylaxis of AF-associated stroke. They also emphasised that individual TTR is a strong indicator of probability for both bleeding and thromboembolic events and should be maintained at 70% or greater.

Since all of these analyses were conducted in non-Asian countries, future analyses will need to show whether the target INR range of 2.0–3.0 can also be used generally for Asian patients to optimise clinical outcome.

As GARFIELD-AF enrols newly diagnosed patients with non-valvular AF, an additional analysis of 4-month rate ratios by TTR for stroke/SE, major bleeding, and all-cause mortality was performed to have a special focus on the initial treatment phase, which is the most critical phase for patients treated with VKA due to the potential prothrombotic effects of these drugs in the early period of therapy [18]. In patients with TTR<65%, the highest rate ratios of stroke/SE and major bleeding were observed during the first 4 months after enrolment and the rates declined in the second and third 4-month periods. Conversely, in patients with TTR≥65% the risk of stroke/SE did not show a trend over time but the risk of major bleeding was highest in the third 4-month period. The latter finding confirms a previous publication reporting an increase of bleeding over time in patients with venous thromboembolism who are anticoagulated with VKA [19]. The authors compared major bleeding in patients with prolonged and short treatment and found major bleeding rates of 2.4% and 0.9%, respectively. They concluded that their analysis of pooled data showed an increase in major bleeding during the entire study period (risk ratio 2.609, 95% CI 1.51 to 4.49, p = 0.0006). The risk of all-cause mortality increased over time in patients with TTR≥65%. This may be due to reasons not related to anticoagulation therapy and will be explored in more detail in future analyses of GARFIELD-AF, after outcome data of all cohorts have become available. As shown in the listing of the corresponding types of stroke in S3 Table, there was an accumulation of ischaemic stroke events for the TTR<65% group during the first 4 months of treatment, whereas the number of haemorrhagic strokes remained unchanged over time, i.e. three events in each 4-month period. Whether this can be attributed to the vulnerable phase of the initiation of VKA, or to patient-related factors, cannot be concluded at the present time and needs further exploration after the termination of this global registry, when the outcome results of all VKA patients from GARFIELD-AF become available. However, these 4-month analyses are in line with the findings of Azoulay et al. [18] that patients initiating warfarin are at an increased risk of stroke during the early period of treatment, supporting the hypothesis that warfarin may induce a transient hypercoagulable state at the start of treatment. Additional studies are needed to confirm these findings.

Our analyses confirmed that poor VKA control correlates with poor clinical outcomes—and this finding is applicable in global patient populations treated under the conditions of different health care systems, although the target INR range of 2.0–3.0 has not been unanimously recommended in all regions and countries participating in GARFIELD-AF. A limitation of this analysis is that TTR was used as a proxy for the quality of patient management. It is a time-dependent variable but it was used as if it was constant over time. Thus, our findings cannot be used to inform patient prognosis nor treatment decisions regarding anticoagulation for individuals with AF [20]. Although the SAMe-TT_2_R_2_ score is helpful in predicting the probability of anticoagulation control with VKAs and clinical outcomes, such studies have only been performed for homogeneous patient populations from single countries [21–24]. These are mainly retrospective [23, 24], single-centre [21, 23, 24] studies, one of which included only patients who were stable on acenocoumarol, with high initial TTR at study entry [21].

In this analysis, the mortality rate was not high; more patients died than had either first stroke/SE or major bleed events. This competing risk of outcomes could have affected the cause-specific rates for stroke/SE and major bleeding, especially in the latter two 4-month intervals. The event rates for these same outcomes were low, especially in the subgroup of patients with TTR≥65% within each 4-month period. Furthermore, there is potential bias in the analysis from the fact that poor TTR is more likely in the presence of concurrent illnesses or procedures (not requiring VKA interruption) that could expose the patient to adverse outcomes, in addition to a poor TTR.

Among the strengths of this paper is that, in contrast to many previous publications on outcomes in patients with AF treated with VKAs, GARFIELD-AF is a global registry reflecting routine clinical practice worldwide, across various health care systems and settings. This is also the first publication of TTR data from a global registry of newly diagnosed patients with AF that reports on a wide range of VKAs, including warfarin, phenprocoumon, acenocoumarol, and others. Furthermore, the data are prospectively collected and potential change of patient care can be timely assessed in the near future.

## Conclusions

These contemporary analyses of VKA control and outcomes in GARFIELD-AF show that patients with poor VKA control have higher risks of stroke/SE, major bleeding, and all-cause mortality than patients with good control, which suggests that overall meticulous VKA management would potentially lead to fewer adverse outcomes. In patients with poor VKA control, the risks of stroke/SE and major bleeding are highest in the first 4 months of treatment. The data also show that the optimal management of VKA for stroke prevention is still a challenge in routine clinical practice. Despite the plethora of publications emphasising the necessity of laboratory-controlled dosing using predefined INR target ranges and the use of VKA for many decades, continuing educational activities are needed to improve stroke prevention in patients with AF.

## Supporting Information

S1 File GARFIELD-AF Registry Investigators. (DOCX)

S2 File Central ethics committees and regulatory authorities. (XLSX)

S1 Table Number of patients enrolled per country. (DOCX)

S2 Table Four-month rate ratios by TTR level. (DOCX)

S3 Table Types of stroke by 4-month intervals in patients with proportion of time in therapeutic range <65% and ≥65%. (DOCX)

## References

[1] Hart RG, Benavente O, McBride R, Pearce LA. Antithrombotic therapy to prevent stroke in patients with atrial fibrillation: a meta-analysis. Ann Intern Med. 1999;131(7):492–501. . 10507957 10.7326/0003-4819-131-7-199910050-00003

[2] Kakkar AK, Mueller I, Bassand JP, Fitzmaurice DA, Goldhaber SZ, Goto S, et al International longitudinal registry of patients with atrial fibrillation at risk of stroke: Global Anticoagulant Registry in the FIELD (GARFIELD). Am Heart J. 2012;163(1):13–9.e1. 10.1016/j.ahj.2011.09.011 . 22172431

[3] Kakkar AK, Mueller I, Bassand JP, Fitzmaurice DA, Goldhaber SZ, Goto S, et al Risk profiles and antithrombotic treatment of patients newly diagnosed with atrial fibrillation at risk of stroke: perspectives from the international, observational, prospective GARFIELD registry. PLoS One. 2013;8(5):e63479 Epub 2013/05/25. 10.1371/journal.pone.0063479 23704912 PMC3660389

[4] Camm AJ, Lip GY, De Caterina R, Savelieva I, Atar D, Hohnloser SH, et al 2012 focused update of the ESC Guidelines for the management of atrial fibrillation: an update of the 2010 ESC Guidelines for the management of atrial fibrillation—developed with the special contribution of the European Heart Rhythm Association. Europace. 2012;14(10):1385–413. 10.1093/europace/eus305 . 22923145

[5] January CT, Wann LS, Alpert JS, Calkins H, Cigarroa JE, Cleveland JC Jr., et al 2014 AHA/ACC/HRS guideline for the management of patients with atrial fibrillation: executive summary: a report of the American College of Cardiology/American Heart Association Task Force on practice guidelines and the Heart Rhythm Society. Circulation. 2014;130(23):2071–104. 10.1161/CIR.0000000000000040 . 24682348

[6] Fitzmaurice DA, Accetta G, Haas S, Kayani G, Lucas Luciardi H, Misselwitz F, et al Comparison of international normalized ratio audit parameters in patients enrolled in GARFIELD-AF and treated with vitamin K antagonists. Br J Haematol. 2016 10.1111/bjh.14084 . 27071942

[7] Rosendaal FR, Cannegieter SC, van der Meer FJ, Briet E. A method to determine the optimal intensity of oral anticoagulant therapy. Thromb Haemost. 1993;69(3):236–9. . 8470047

[8] NICE. Atrial fibrillation: management2014 16 Mar 2016. Available from: http://www.nice.org.uk/guidance/cg180/resources/guidance-atrial-fibrillation-the-management-of-atrial-fibrillation-pdf.

[9] Le Heuzey JY, Ammentorp B, Darius H, De Caterina R, Schilling RJ, Schmitt J, et al Differences among western European countries in anticoagulation management of atrial fibrillation. Data from the PREFER IN AF registry. Thromb Haemost. 2014;111(5):833–41. 10.1160/TH13-12-1007 . 24651882

[10] JCS Joint Working Group. Guidelines for Pharmacotherapy of Atrial Fibrillation (JCS 2013). Circ J. 2014;78(8):1997–2021. . 24965079 10.1253/circj.cj-66-0092

[11] Ogawa S, Aonuma K, Tse H-F, Huang D, Huang J-L, Kalman J, et al The APHRS's 2013 statement on antithrombotic therapy of patients with nonvalvular atrial fibrillation. Journal of Arrhythmia. 2013;29(3):190–200.

[12] Federatie van Nederlandse Trombosediensten. De kunst van het doseren: Richtlijn, leidraad en informatie voor het doseren van vitamine K-antagonisten2015 16 May 2016;. Available from: http://www.elkerliek.nl/elkerliek.nl/specialismen/AKL/De%20kunst%20van%20het%20doseren.pdf.

[13] Oh S, Goto S, Accetta G, Angchaisuksiri P, Camm AJ, Cools F, et al Vitamin K antagonist control in patients with atrial fibrillation in Asia compared with other regions of the world: Real-world data from the GARFIELD-AF registry. Int J Cardiol. 2016;223:543–7. 10.1016/j.ijcard.2016.08.236 . 27552578

[14] Mearns ES, White CM, Kohn CG, Hawthorne J, Song JS, Meng J, et al Quality of vitamin K antagonist control and outcomes in atrial fibrillation patients: a meta-analysis and meta-regression. Thromb J. 2014;12:14 10.1186/1477-9560-12-14 25024644 PMC4094926

[15] Hylek EM, Skates SJ, Sheehan MA, Singer DE. An analysis of the lowest effective intensity of prophylactic anticoagulation for patients with nonrheumatic atrial fibrillation. N Engl J Med. 1996;335(8):540–6. 10.1056/NEJM199608223350802 . 8678931

[16] Gallagher AM, Setakis E, Plumb JM, Clemens A, van Staa TP. Risks of stroke and mortality associated with suboptimal anticoagulation in atrial fibrillation patients. Thromb Haemost. 2011;106(5):968–77. 10.1160/TH11-05-0353 . 21901239

[17] Björck F, Renlund H, Lip GYH, Wester P, Svensson PJ, Själander A. Outcomes in a Warfarin-Treated Population With Atrial Fibrillation. JAMA Cardiology. 2016 10.1001/jamacardio.2016.0199 27437888

[18] Azoulay L, Dell'Aniello S, Simon TA, Renoux C, Suissa S. Initiation of warfarin in patients with atrial fibrillation: early effects on ischaemic strokes. Eur Heart J. 2014;35(28):1881–7. Epub 2013/12/20. 10.1093/eurheartj/eht499 . 24353282

[19] Middeldorp S, Prins MH, Hutten BA. Duration of treatment with vitamin K antagonists in symptomatic venous thromboembolism. Cochrane Database Syst Rev. 2014;8:CD001367 10.1002/14651858.CD001367.pub3 . 25092359 PMC7074008

[20] Alexander JH, Thomas LE. Using Data to Guide Anticoagulation in Patients With Atrial Fibrillation: Does the Analysis Fit the Question? JAMA Cardiol. 2016;1(2):121–2. 10.1001/jamacardio.2016.0198 . 27437881

[21] Gallego P, Roldan V, Marin F, Galvez J, Valdes M, Vicente V, et al SAMe-TT2R2 score, time in therapeutic range, and outcomes in anticoagulated patients with atrial fibrillation. Am J Med. 2014;127(11):1083–8. Epub 2014/05/27. 10.1016/j.amjmed.2014.05.023 . 24858062

[22] Lip GY, Haguenoer K, Saint-Etienne C, Fauchier L. Relationship of the SAMe-TT(2)R(2) score to poor-quality anticoagulation, stroke, clinically relevant bleeding, and mortality in patients with atrial fibrillation. Chest. 2014;146(3):719–26. 10.1378/chest.13-2976 . 24722973

[23] Abumuaileq RR, Abu-Assi E, Raposeiras-Roubin S, Lopez-Lopez A, Redondo-Dieguez A, Alvarez-Iglesias D, et al Evaluation of SAMe-TT2R2 risk score for predicting the quality of anticoagulation control in a real-world cohort of patients with non-valvular atrial fibrillation on vitamin-K antagonists. Europace. 2015;17(5):711–7. 10.1093/europace/euu353 . 25662984

[24] Chan PH, Hai JJ, Chan EW, Li WH, Tse HF, Wong IC, et al Use of the SAMe-TT2R2 Score to Predict Good Anticoagulation Control with Warfarin in Chinese Patients with Atrial Fibrillation: Relationship to Ischemic Stroke Incidence. PLoS One. 2016;11(3):e0150674 10.1371/journal.pone.0150674 27010633 PMC4807017

