# Supplementary material for: Quality of Vitamin K Antagonist Control and 1-Year Outcomes in Patients with Atrial Fibrillation: A Global Perspective from the GARFIELD-AF Registry
Source: PLoS One. 2016 Oct 28;11(10):e0164076. doi: 10.1371/journal.pone.0164076 (PMC5085020; doi:10.1371/journal.pone.0164076)
Supplement: S1 Table — (DOCX) [file pone.0164076.s003.docx]

| **Region** | **Country** | **n (%) (N=9934)** |
| --- | --- | --- |
| **Europe** |  |  |
|  | Austria | 92 (0.9) |
|  | Belgium | 106 (1.1) |
|  | Czech Republic | 435 (4.4) |
|  | Denmark | 148 (1.5) |
|  | Finland | 226 (2.3) |
|  | France | 283 (2.8) |
|  | Germany | 616 (6.2) |
|  | Hungary | 424 (4.3) |
|  | Italy | 831 (8.4) |
|  | Netherlands | 650 (6.5) |
|  | Norway | 58 (0.6) |
|  | Poland | 459 (4.6) |
|  | Russia | 70 (0.7) |
|  | Spain | 790 (8.0) |
|  | Sweden | 404 (4.1) |
|  | Switzerland | 2 (0.0) |
|  | Ukraine | 154 (1.6) |
|  | United Kingdom | 1092 (11.0) |
| **Asia** |  |  |
|  | China | 71 (0.7) |
|  | India | 28 (0.3) |
|  | Japan | 506 (5.1) |
|  | Korea | 915 (9.2) |
|  | Singapore | 57 (0.6) |
|  | Thailand | 286 (2.9) |
|  | Turkey | 9 (0.1) |
|  | United Arab Emirates | 27 (0.3) |
| **North America** |  |  |
|  | Canada | 157 (1.6) |
|  | United States | 63 (0.6) |
| **Latin America** |  |  |
|  | Argentina | 107 (1.1) |
|  | Brazil | 86 (0.9) |
|  | Chile | 327 (3.3) |
|  | Mexico | 68 (0.7) |
| **Rest of the world** |  |  |
|  | Australia | 195 (2.0) |
|  | Egypt | 7 (0.1) |
|  | South Africa | 185 (1.9) |
