# Supplementary material for: Quality of Vitamin K Antagonist Control and 1-Year Outcomes in Patients with Atrial Fibrillation: A Global Perspective from the GARFIELD-AF Registry
Source: PLoS One. 2016 Oct 28;11(10):e0164076. doi: 10.1371/journal.pone.0164076 (PMC5085020; doi:10.1371/journal.pone.0164076)
Supplement: S2 Table — (DOCX) [file pone.0164076.s004.docx]

|  |  | **TTR<65%** | | **TTR≥65%** | | **Total** | |
| --- | --- | --- | --- | --- | --- | --- | --- |
|  |  | **Rate ratio** | **95% CI** | **Rate ratio** | **95% CI** | **Rate ratio** | **95% CI** |
| **Stroke/SE** | 1^st^ to 4^th^ months | 1.32 | 0.91 to 1.91 | 1.08 | 0.54 to 2.16 | 1.26 | 0.91 to 1.75 |
|  | 5^th^ to 8^th^ months | 1.11 | 0.73 to 1.66 | 1.08 | 0.54 to 2.17 | 1.10 | 0.77 to 1.56 |
|  | 9^th^ to 12^th^ months | 0.55 | 0.31 to 1.00 | 0.83 | 0.37 to 1.85 | 0.62 | 0.39 to 1.00 |
|  | Total | 1 | (ref) | 1 | (ref) | 1 | (ref) |
| **Major bleeding** | 1^st^ to 4^th^ months | 1.37 | 0.97 to 1.94 | 0.86 | 0.45 to 1.66 | 1.22 | 0.90 to 1.65 |
|  | 5^th^ to 8^th^ months | 1.01 | 0.67 to 1.52 | 0.38 | 0.14 to 1.02 | 0.81 | 0.56 to 1.18 |
|  | 9^th^ to 12^th^ months | 0.59 | 0.35 to 1.02 | 1.77 | 1.12 to 2.81 | 0.97 | 0.68 to 1.37 |
|  | Total | 1 | (ref) | 1 | (ref) | 1 | (ref) |
| **All-cause mortality** | 1^st^ to 4^th^ months | 0.84 | 0.65 to 1.09 | 0.70 | 0.41 to 1.21 | 0.82 | 0.65 to 1.03 |
|  | 5^th^ to 8^th^ months | 1.00 | 0.79 to 1.27 | 0.76 | 0.45 to 1.28 | 0.95 | 0.77 to 1.18 |
|  | 9^th^ to 12^th^ months | 1.17 | 0.93 to 1.46 | 1.55 | 1.07 to 2.24 | 1.24 | 1.03 to 1.51 |
|  | Total | 1 | (ref) | 1 | (ref) | 1 | (ref) |

CI, confidence interval; SE, systemic embolism; TTR, time in therapeutic range.
