# Supplementary material for: Quality of Vitamin K Antagonist Control and 1-Year Outcomes in Patients with Atrial Fibrillation: A Global Perspective from the GARFIELD-AF Registry
Source: PLoS One. 2016 Oct 28;11(10):e0164076. doi: 10.1371/journal.pone.0164076 (PMC5085020; doi:10.1371/journal.pone.0164076)
Supplement: S3 Table — (DOCX) [file pone.0164076.s005.docx]

|  | **TTR<65%** | | | | **TTR≥65%** | | | |
| --- | --- | --- | --- | --- | --- | --- | --- | --- |
|  | **1^st^ to 4^th^ months** | **5^th^ to 8^th^ months** | **9^th^ to 12^th^ months** | **Total** | **1^st^ to 4^th^ months** | **5^th^ to 8^th^ months** | **9^th^ to 12^th^ months** | **Total** |
| Stroke/SE | 36 | 29 | 17 | 82 | 8 | 10 | 8 | 26 |
| Stroke (not including SE) | 34 | 24 | 17 | 75 | 8 | 6 | 6 | 18 |
| Primary ischaemic | 23 | 17 | 9 | 49 | 5 | 6 | 1 | 12 |
| Of which secondary haemorrhagic | *1* | *1* | *1* | *3* | *0* | *1* | *0* | *1* |
| Primary intracerebral haemorrhage | 6 | 1 | 5 | 12 | 2 | 0 | 4 | 6 |
| Undetermined* | 5 | 6 | 3 | 14 | 1 | 0 | 1 | 2 |

*Includes patients with unknown types of stroke and those with both primary ischaemic and primary intracerebral haemorrhagic strokes.

SE, systemic embolism; TTR, time in therapeutic range.
